# Growth, Mechanical, Thermal and Spectral Properties of Cr^3+^∶MgMoO_4_ Crystal

**DOI:** 10.1371/journal.pone.0030327

**Published:** 2012-01-24

**Authors:** Lingyun Li, Yisheng Huang, Lizhen Zhang, Zhoubin Lin, Guofu Wang

**Affiliations:** 1 State Key Laboratory of Optoelectronics Material Chemistry and Physics, Fujian Institute of Research on the Structure of Matter, Chinese Academy of Sciences, Fuzhou, Fujian, China; 2 Graduate School of Chinese Academy of Sciences, Beijing, China; University of Nottingham, United Kingdom

## Abstract

This paper reports the growth, mechanical, thermal and spectral properties of Cr^3+^∶MgMoO_4_ crystals. The Cr^3+^∶MgMoO_4_ crystals with dimensions up to 30 mm×18 mm×14 mm were obtained by TSSG method. The absorption cross-sections of ^4^A_2_→^4^T_1_ and ^4^A_2_→^4^T_2_ transitions are 12.94×10^−20^ cm^2^ at 493 nm and 7.89×10^−20^ cm^2^ at 705 nm for E//**N_g_**, respectively. The Cr^3+^∶MgMoO_4_ crystal shows broad band emission extending from 750 nm to 1300 nm with peak at about 705 nm. The emission cross-section with FWHM of 188 nm is 119.88×10^−20^ cm^2^ at 963 nm for E//**N_g_**. The investigated results showed that the Cr^3+^∶MgMoO_4_ crystal may be regarded as a potential tunable laser gain medium.

## Introduction

Tunable solid-state lasers have a wide field of applications in medicine, military, ultra short pulse generation and communication [1], [2]. Since 1960 many Cr^3+^-doped tunable laser crystals have been investigated, such as BeAl_2_O_4_, LiCaAlF_6_, Be_3_Al_2_(SiO_3_)_6_, GdScGa-Garnet and LaSc(BO_3_)_4_ [3]–[9]. Some of them have been became commercial material, such as Cr∶LiSrAlF_6_. Recently, research is focusing on the tunable solid-state laser crystals in visible and near infrared spectrum region for flash lamping and diode laser pumping. The Cr^3+^-doped molybdate crystals have gained interest because they have broad emission bands and larger absorption and emission cross-sections [10]–[12].

Metal molybdates of the general formula AMoO_4_ (A = Mg, Cd, Pb, Zn and Ca) have been attracted much attention owing to their important application of optoelectronic devices [13]–[16]. MgMoO_4_ is a member of this family, it belongs to monoclinic system with C2/m space group and cell parameters a = 10.273, b = 9.288, c = 7.025, β = 106.96°, z = 8. Recently, the MgMoO_4_ and Yb^3+^-doped MgMoO_4_ crystal were reported as a cryogenic phonon-scintillation detector [13]. The spectral properties of the Cr^3+^∶MgMoO_4_ crystal were reported such as crystal field strength and Racah parameters [17]. In this paper we further report the growth, mechanical, thermal and polarized spectral characteristics of the Cr^3+^∶MgMoO_4_ crystal.

## Materials and Methods

### 1. Crystal Growth

**Figure 1:**
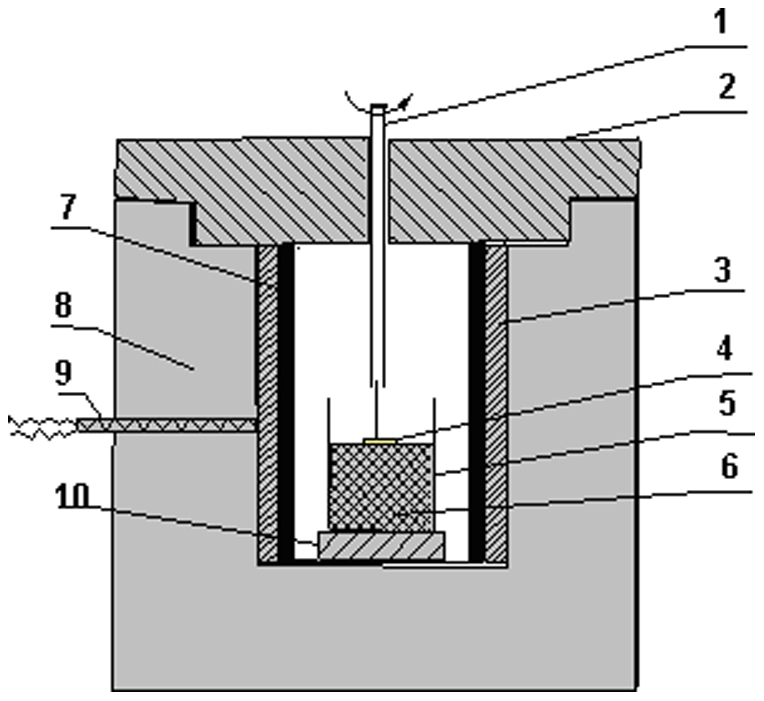
Schematic diagram of crystal growth apparatuses: (1) seed holder; (2) furnace cover; (3) Nickel- Chromium resistant wire; (4) seed; (5) crucible; (6) melt; (7) Al_2_O_3_ tube; (8) thermal insulation material ; ( 9) thermocouple; (10) crucible holder.

**Figure 2:**
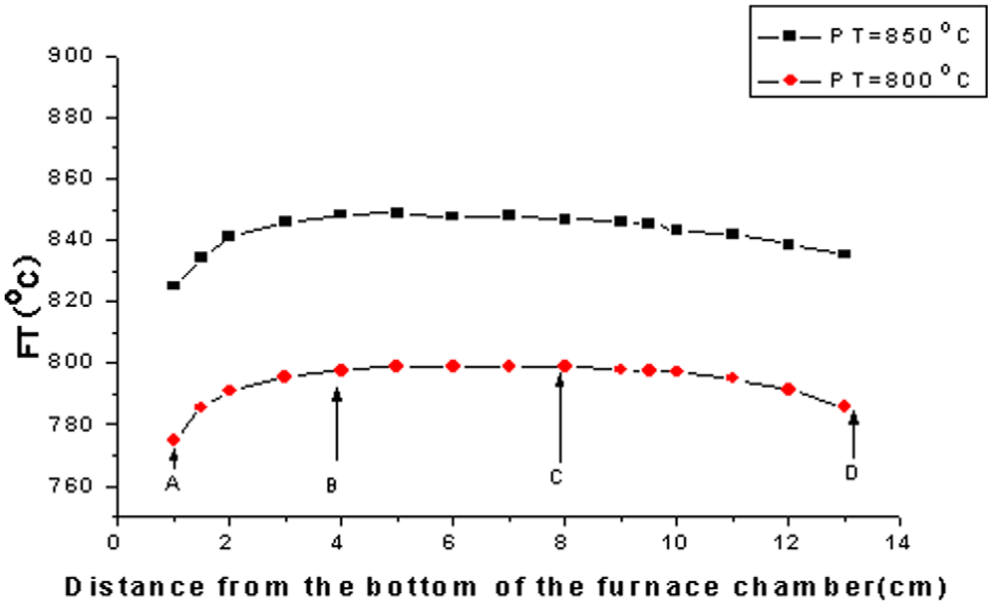
Longitudinal temperature field in the furnace chamber(FT = temperature in the chamber, PT = programmed temperature).

Since the MgMoO_4_ crystal melts congruently at 1320°C, it can generally be grown by the Czochralski method, i. e. pulling directly from melt of the MgMoO_4_ crystal. However the MgMoO_4_ crystals with good quality were difficulty obtained because of the strong evaporation of MoO_3_ component under high temperature [18], [19]. Therefore, in order to reduced the growth temperature we selected the top seeded solution growth (TSSG) method to grow the Cr^3+^∶MgMoO_4_ crystals. The Cr^3+^∶MgMoO_4_ crystals were grown by the top seeded solution growth (TSSG) method from a flux of K_2_Mo_2_O_7_. The chemicals used were MgO, K_2_CO_3_, Mo_2_O_3_ and Cr_2_O_3_ with purity of 99.99%. The crystal growth was carried out in a vertical tubular furnace with a nickel-chrome wire as the heating element, as shown in Fig. 1. An AL-708 controller with a Pt-PtRh thermocouple controlled the furnace temperature and the cooling rate [20]. The temperature gradient in the furnace chamber was measured before performing of crystal growth, as shown in Fig. 2. The longitudinal temperature field in the furnace chamber could be divided into three parts: the flat zone ranging from B to C and the gradient zone ranging from A to B and C to D. The crystal growth was performed in the flat temperature zone in the furnace, which is available to grow large size crystal.

**Figure 3:**
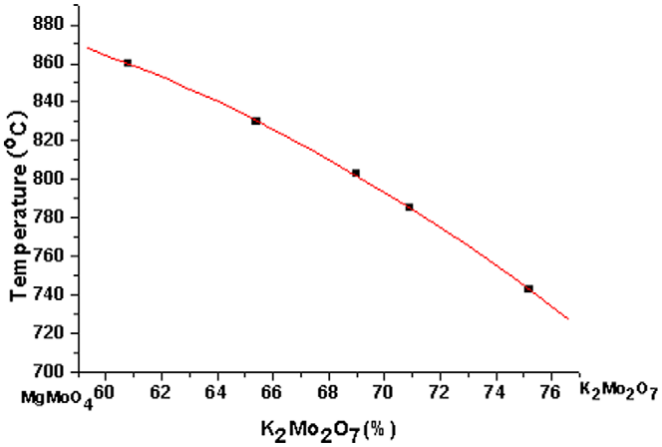
Solubility curve of MgMoO_4_ in MgMoO_4_-K_2_Mo_2_O_7_ system.

In order to select the suitable composition of the solution, the solubility curve of the MgMoO_4_ in the solution of MgMoO_4_-K_2_Mo_2_O_7_ was determined by the trial seeding method. The saturation temperatures were determined for various compositions in the range of 60–75 mol% by adjusting the temperature of the solution until a trial seeding showed no change in weight or surface micro topography after 3–4 h immersion. Fig. 3 shows the solubility curve of the MgMoO_4_ in the solution.

**Figure 4:**
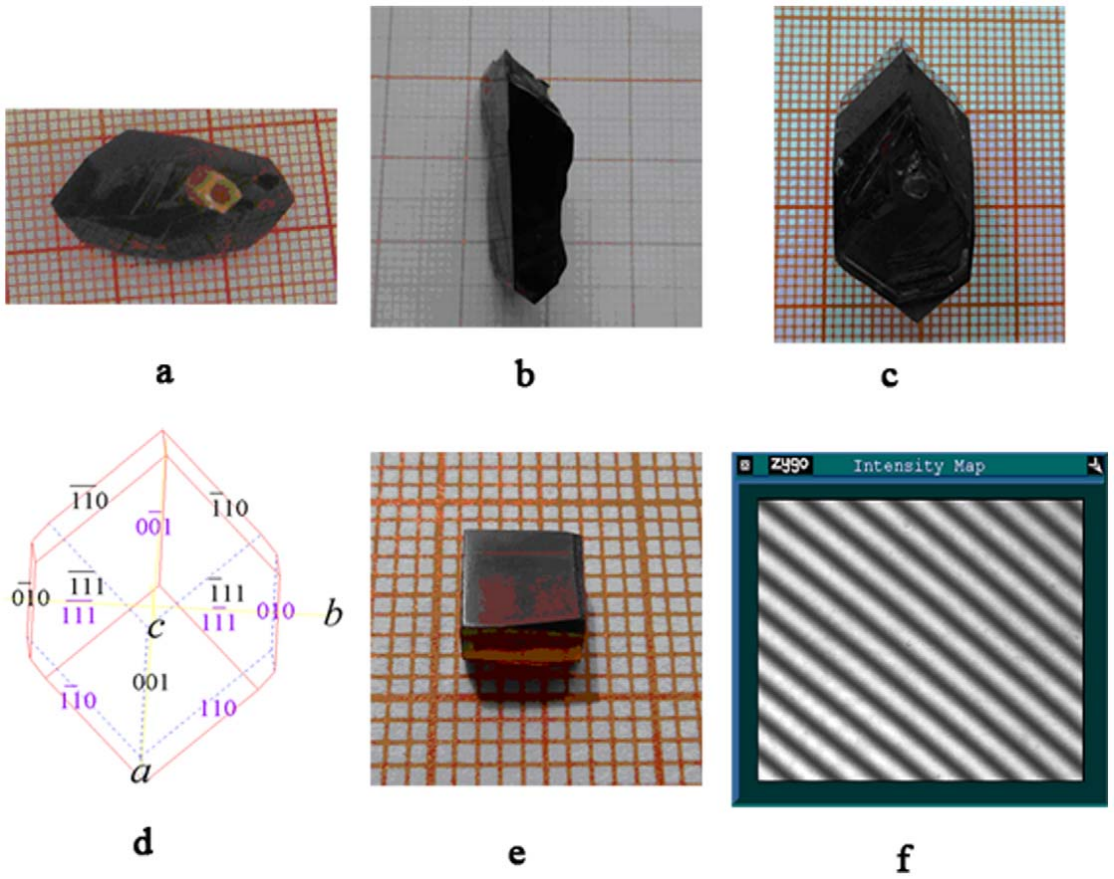
As-grown Cr^3+^∶ MgMoO_4_ : (a) crystal grown along [010] direction; (b) crystal grown along [ 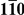
 **]; (c) crystal grown along [001] direction; (d) predicted hexagonal shape of Cr^3+^∶MgMoO_4_); (e) a cut sample with free inclusion ; (f) interference fringe of crystal.** (where the color is reflecting light).

The crystal growth was performed by TSSG method. The procedure is as follows: firstly, the starting materials of 2 at% Cr^3+^-doped MgMoO_4_ and K_2_Mo_2_O_7_ were weighed according to the ratio of MgMoO_4_∶ K_2_Mo_2_O_7_ = 2∶3 mol. The weighed materials were mixed and put into the platinum crucible with dimension of Ø50 mm×60 mm. The full charged crucible was placed into the furnace and kept at 950°C for 48 h to make the solution melt completely and homogeneously. Secondly, a platinum wire was as seed crystal was soaked into the solution, and the temperature was cooled down from 950°C to 835°C at a cooling rate of 2°C/d. Then, the crystals grown on the platinum wire were drawn out of the solution surface and cooled down to room temperature at a cooling rate of 20°C/h. Finally, after obtained small crystals, a seed cut from the as-obtained crystal was used to grow large size crystals. The saturation temperature of the solution was exactly determined to be 865°C by repeated seeding. Then the seed was dipped into the solution at a temperature 20°C above saturation temperature and was kept at this temperature for 20 min to dissolve the surface of the seed. The crystals were grown at a cooling rate of 1°C/d and rotated at a rotating rate of 15 rpm in the range of 865∼835°C. In comparison with the Czochralski method, the starting growth temperature was reduced from 1320°C to 865°C, which greatly reduced the evaporation of MoO_3_ component. When the growth process ended, the crystals were pulled out of the solution and cooled to room temperature at a cooling rate of 20°C/h. The grown crystals with few inclusions were shown in Fig. 4, in which the Cr^3+^∶MgMoO_4_ crystals were grown along [001], [010] and [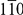] directions, respectively. The maximum size is up to 30 mm×18 mm×14 mm. The morphology of the grown crystals depends on the growing direction. The morphology of the crystal grown along [001] direction appeared a regular hexagonal shape with families of crystal planes {001}, {110} and {111}, as shown in Fig. 4(c and d). A sample with dimensions of 5.39 mm×4.82 mm×3.92 mm and free inclusion was cut from the as-grown crystal (Fig. 4(e)). The optical homogeneity of the Cr^3+^∶MgMoO_4_ crystal was determined to be 3.9×10^−5^ using Tyman-Green optical interferometer, as shown in Fig. 4(f). This result shows that the grown crystal has good quality.

The concentration of Cr^3+^ ion in the Cr^3+^∶MgMoO_4_ crystal was determined to be 0.48 at% by inductively coupled plasma atomic emission spectrometry (ICP-AES). Then, the segregation coefficient of Cr^3+^ ion in crystal is defined as following formula:
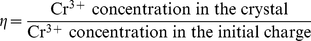
(1)Thus, the segregation coefficient of Cr^3+^ ion in the Cr^3+^∶MgMoO_4_ crystal is 0.24. The X-ray powder diffraction pattern of the Cr^3+^∶MgMoO_4_ was collected by MiniFlex II powder diffractometer with Cu K_α_ radiation, and the result was consistent with that reported by V.V. Bakakin [21].

### 2. Mechanical and Thermal Properties

The hardness is an important mechanical property of the crystal materials. The hardness of the Cr^3+^∶MgMoO_4_ crystal was measured using a Vickers microhardness tester (WILSON-WOLPERT 401MVA™) which equipped with a diamond square pyramid indenter attached to an incident-light microscope. Three samples with [100], [010] and [001] directions were cut from the as-grown Cr^3+^∶MgMoO_4_ crystal and polished for the test. The load applied for indenting was 200 kg and the indentation time was kept at 10 s for all samples. The value of the Vickers microhardness *HV* is calculated using the following expression
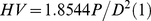
(2)where *P* is the applied load and *D* is the diagonal length of the indentation.

For a crystal with well-defined cracks, the resistance to fracture indicates the toughness of a material. According to Ref. 20, fracture toughness *K_c_* is dependent on the ratio of *c*/*a*, where *c* is the crack length and *a* is the half-diagonal length of the square indentation. When *c*/*a*≤2.5, the cracks have the Palmqvist's configuration, *K_c_* is calculated using the equation
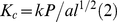
(3)where *l = c−a* is the mean Palmqvist's crack length, the constant *k* is 1/7 for the Vickers indenter.

The brittleness index *B_i_* is calculated using the relation
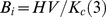
(4)


The calculated results are listed in Table 1.

**Table 1:** Results of the mechanical properties of Cr^3+^∶ MgMoO_4_ crystal.

Direction	*P*(kg)	*a*(µm)	*c*(µm)	*D*(µm)	*HV*(10^3^ MPa)	*K_c_*(10^3^ MPa·m^1/2^)	*B_i_*(m^−1/2^)
[100]	200	18.39	29.56	36.79	273.96 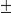 0.15	4.65	58.93±0. 04
[010]	200	17.07	30.95	34.15	317.95 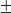 0.19	4.49	70.77±0.06
[001]	200	19.05	36.53	38.1	255.44 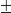 0.13	3.59	1.21±0.04

**Figure 5:**
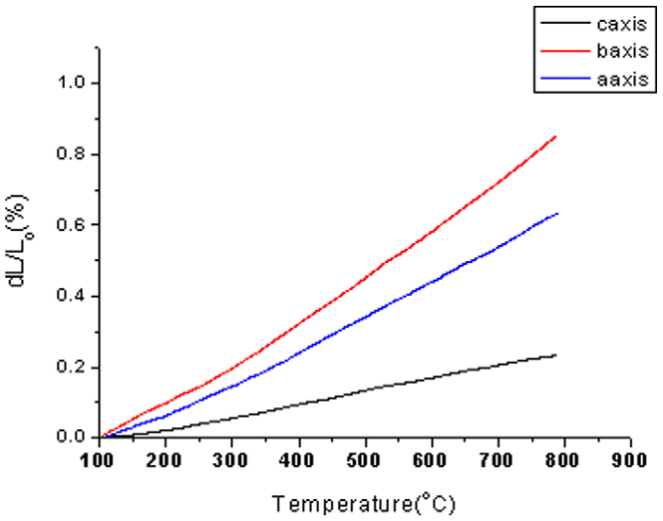
Thermal expansion of Cr^3+^∶ MgMoO_4_ crystal.

The thermal expansion of crystal is another important thermal factor for the crystal. Since the Cr^3+^∶ MgMoO_4_ crystal with monoclinic system is of anisotropy, three samples with dimensions of 6.0 mm×6.0 mm×20 mm used for thermal expansion coefficient measurement were cut from the Cr^3+^∶ MgMoO_4_ crystal along ***a***-axis, ***b***-axis and ***c***-axis, respectively. The thermal expansion coefficients were measured using a DIL 402PC type thermal expansion dilatometer instrument at a heating rate of 10°C/min and the result in the range of 100∼800°C. Fig. 5 shows the linear expansion versus the temperature. The linear thermal expansion coefficient is defined as:
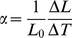
(5)where *L_0_* is the initial length of the sample at room temperature,*ΔL* is the change in length when temperature changes *ΔT*. The thermal expansion coefficient was calculated from the slope of the linear fitting of the linear relation between *ΔL/L* and temperature. The thermal expansion coefficients were calculated and listed in Table 2. The results show that the thermal expansion coefficient exhibits strongly direction dependence, the thermal expansion coefficient along ***b***-axis is 3.3 times than that along ***c***-axis.

**Table 2:** Thermal expansion coefficient of Cr^3+^∶ MgMoO_4_ crystal.

	*a*-axis	*b*-axis	*c*-axis
Thermal expansion Coefficient(10^−6^/K)	10.10	12.26	3.71

### 3. Spectral Properties

**Figure 6:**
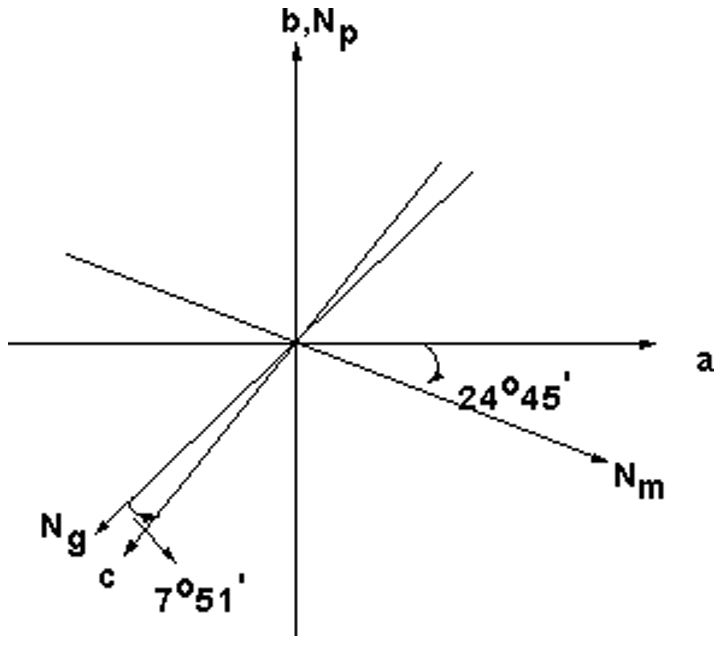
Angular relation between the optical indicatrix axes and the crystallographic axes of MgMoO_4_ crystal.

Since the MgMoO_4_ crystal belongs to monoclinic system, there are three refractive indices along the optical indicatrix axis (**N_g_**, **N_m_**, **N_p_**) which do not coincide with the crystallographic axes (***a***, ***b***, ***c***). **N_g_** and **N_m_** are located in the ***ac*** plane, while **N_p_** is parallel to ***b***-axes. The angular relation between the two sets axes of MgMoO_4_ crystal was determined by a polarizing microscope. Fig. 6 shows the relative orientation of the optical indicatrix axis (**N_g_**, **N_m_**, **N_p_**) relative to the crystallographic axes (***a***, ***b***, ***c***) of MgMoO_4_, **N_m_** was located at about 7°51′ to ***–c*** axis, therefore **N_g_** was located at about 24°25′ to ***a*** axis.

Based on the results obtained above, a sample of the Cr^3+^∶MgMoO_4_ crystal with dimension of 5.39 mm×4.82 mm×3.92 mm was cut from as-grown crystal and polished for the spectroscopic experiments,as shown in Fig. 4(e). The edges of cuboid were parallel to the optical indicatrix axis **N_g_**, **N_m_**, **N_p_**, respectively. The polarized absorption spectrum was measured using a Perkin-Elmer UV-VIS-NIR spectrometer (Lambda-900) in the range of 300–1100 nm at room temperature. The polarized fluorescence spectra were measured using the Edinburgh Analysis Instruments FLS920 spectrophotometer with Xenon lamp as light source. The fluorescence lifetime was measured by by Lifespec-ps system of Edinburgh Instruments Ltd. The light source is continuous tunable picosecond pulsed Ti: sapphire (Tsunami+GWU). In experiment of lifetime measurement, the pulse duration of the incident light is 2∼100 ps, the time resolution of the MCP-PMT detector is about 50 ps, the resolution of the monochromater is 0.5∼2 nm, and the signal-to-noise ratio of Lifespec-ps system is 6000∶1. The wavelength of the excited light is 700 nm, and the detection wavelength is 820 nm.

**Figure 7:**
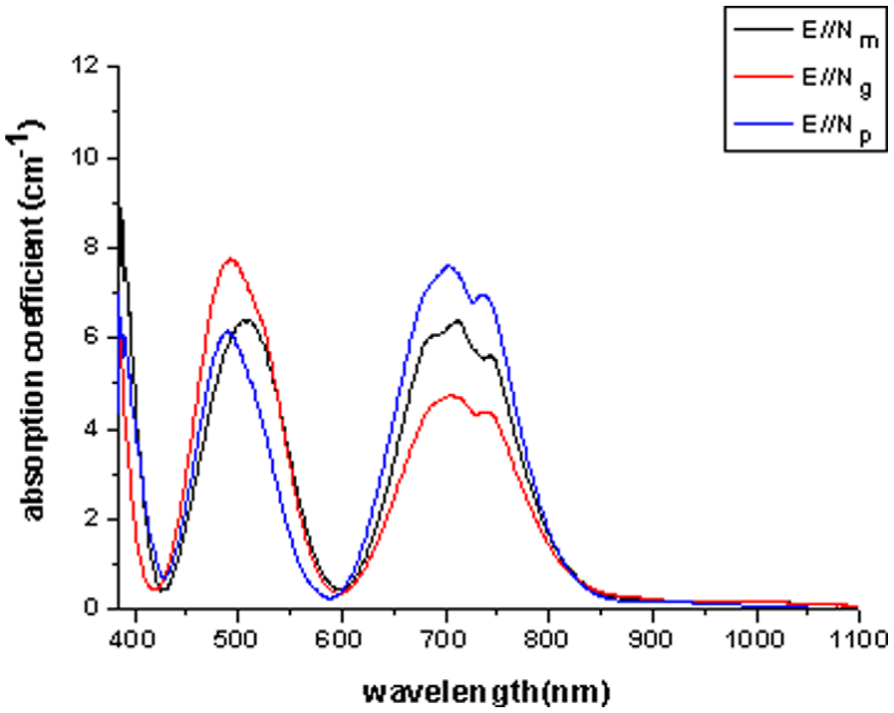
Polarized absorption spectrum of Cr^3+^∶MgMoO_4_ crystal at room temperature.

Fig. 7 shows the polarized absorption spectra of the Cr^3+^∶MgMoO_4_ crystal measured at room temperature. The spectrum consists of two broad absorption bands centered at about 492 nm and 703 nm, corresponding to the electron-vibronic transitions from the ^4^A_2_ ground state to the excited ^4^T_2_ and ^4^T_1_ states. The dip presented at 726 nm on the low energy band is caused by Fano-type antiresonance due to the spin forbidden transition from ^4^A_2_ to ^2^E and the R-lines were not observed [22], [23]. The structure of the absorption band was characteristic of the Cr^3+^ ion in a weak crystal field as well as the absorption spectrum of the Cr^3+^∶LiSrAlF_6_ and Cr^3+^∶LaSc(BO_3_)_4_ [5], [8]. The absorption cross-sections *σ*_a_ were determined to be 12.9×10^−20^ cm^2^ at 491 nm and 7.89×10^−20^ at 705 nm for E//**N_g_**, respectively.

**Figure 8:**
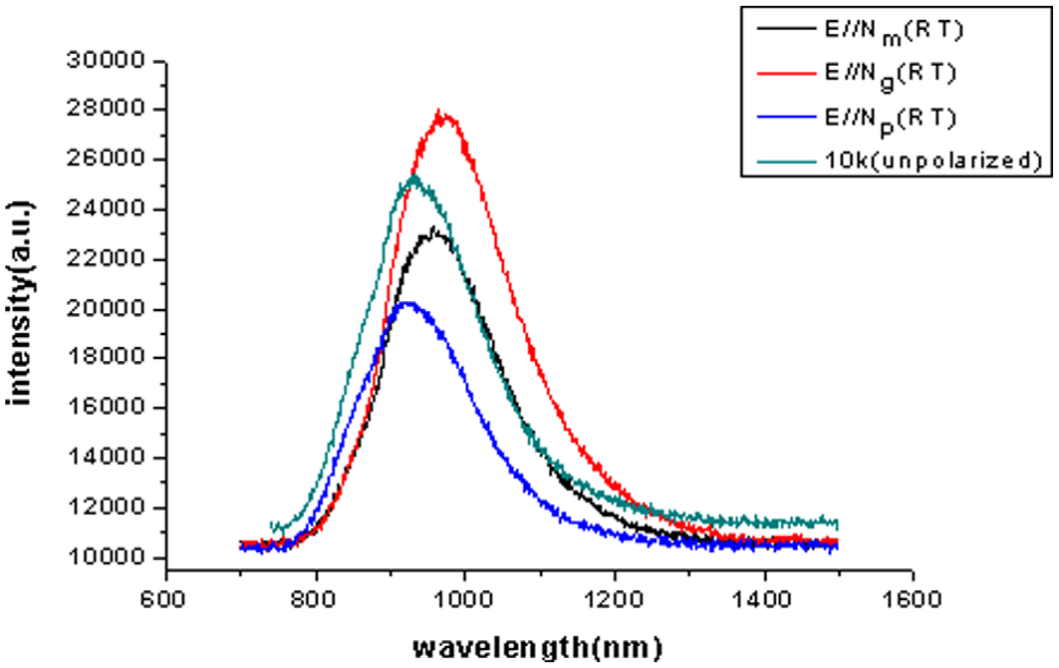
Polarized and unpolarized fluorescence spectra of Cr^3+^∶MgMoO_4_ crystal excited with 700 nm radiation at room temperature and 10K.

Fig. 8 presented the polarized and unpolarized fluorescence spectra measured at room temperature and 10K. It is shown that the main feature of the fluorescence spectra is a broad band extending from 750 nm to 1300 nm, corresponding to the transition from ^4^T_2_ excited level to ^4^A_2_ ground level. Even at 10K it is still a broad emission band. The luminescence spectra of Cr^3+^∶MgMoO_4_ crystals are strongly polarized at room temperature. The broadest emission band was observed with a peak at 963 nm with a full width at half maximum (FWHM) of 188 nm for E//**N_g_**. Such broad absorption and emission bands were caused by the structure of the MgMoO_4_ crystal, except for its broad and emission transitions of the Cr^3+^ ions. It is reason that the structure of the MgMoO_4_ crystal consists of two types of MgO_6_ tetrahedra [24], the Cr^3+^ ions occupied the different Mg^2+^ sites in the two types of MgO_6_ tetrahedra when the Cr^3+^ ions were doped into the MgMoO_4_ crystal and replaced the Mg^2+^ ions. In other word, the Cr^3+^ ions occupied the two luminous centers, which results in broad absorption and emission bands.

The absorption and fluorescence spectra of the Cr^3+^∶MgMoO_4_ crystal indicated that the Cr^3+^ ions in the Cr^3+^∶MgMoO_4_ crystal occupied a weak-field site, in which the ^4^T_2_ level is below the ^2^E level. According to Tanabe-Sugano diagram [25], the signification of Cr^3+^ ions occupying strong-field or weak-field sites is immediately apparent from the luminescence spectrum. In the strong-field the luminescence spectrum consists of broadband emission of ^4^T_2_→^2^A_2_ transition and sharp line emission of ^2^E_2_→^2^A_2_ transition. The weak-field sites in the Cr^3+^∶MgMoO_4_ crystal give rise to the Cr^3+^ luminescence in the ^4^T_2_→^2^A_2_ transition band alone, even at 10K dominant feature of photoluminescence spectrum is still broadband emission, which is available for tunable laser crystal. The fluorescence lifetime 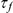 was determined to be 1 μs measured at room temperature. Since the radiation lifetime is mainly derived from the parity-forbidden ^4^T_2_→^2^A_2_ transition with short lifetime in the weak-field, the Cr^3+^∶MgMoO_4_ crystal has a very short fluorescence lifetime.

The emission cross-section *σ*_e_ was calculated using the formula [26]

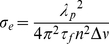
(6)where λ is the wavelength of the emission peak, *n* is the refractive index of the Cr^3+^∶MgMoO_4_ crystal, which was determined by the method of minimum deviation and the value was listed in Table 3, and 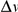 the frequency of FWHM. The 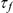 is the fluorescence lifetime, which was determined to be 1 µs. Thus, the emission cross-section of ^4^T_2_→^4^A_2_ transition is 119.88×10^−20^ cm^2^ at 963 nm for E//**N_g_**.

**Table 3:** Refractive index value of the wavelength at emission peak.

Wavelength(nm)	919(E//N_p_)	956(E//N_m_)	963(E//N_g_)
Refractive index	1.746	1.787	1.819

## Results and Discussion

The Cr^3+^∶MgMoO_4_ crystals with dimensions up to 30 mm×18 mm×14 mm were obtained by TSSG method. The mechanical and thermal properties and polarized optical characteristic of the Cr^3+^∶MgMoO_4_ were investigated. The thermal expansion coefficient of the Cr^3+^∶MgMoO_4_ crystal along ***c***-axis is smaller than that of the other directions. The investigated results of spectral properties of the Cr^3+^∶MgMoO_4_ crystal showed that its absorption and emission spectra exhibit strong polarized and depend on the optical indicatrix axis **N_g_**, **N_m_**, **N_p_** at room temperature. The Cr^3+^∶MgMoO_4_ crystal has large absorption cross-section at about 705 nm, which is available for the diode laser pumping. The Cr^3+^∶MgMoO_4_ crystal exhibits a broad band emission extending from 750 nm to 1300 nm with peak at about 705 nm. The emission cross-section with FWHM of 188 nm is 119.88×10^−20^ cm^2^ at 963 nm for E//**N_g_**. In comparison with other Cr^3+^ doped materials (Table 4), the Cr^3+^∶MgMoO_4_ crystal has large emission cross-section and FWHM of the fluorescence. The fluorescence lifetime 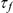 was determined is 1 μs, which is available to apply to short pulse laser. To sum up above the results, the conclusion was drawn that the Cr^3+^∶MgMoO_4_ crystal may be regarded as a potential tunable laser gain medium.

**Table 4:** Spectral parameters of Cr^3+^∶ MgMoO_4_ crystal and other tunable laser crystals.

Crystals		^4^A_2_→^4^T_1_		^4^A_2_→^4^T_2_			^4^T_2_→^4^A_2_		Ref.
	λ(nm)	*σ*_e_(10^−20^ cm^2^)	λ(nm)	*σ*_e_(10^−20^ cm^2^)	λ(nm)	FWHM	*σ*_e_(10^−20^ cm^2^)	τ_f_(µs)	
Cr∶BeAl_2_O_4_	420	10.0	600	20.0	750	-	0.5	260	[3]
Cr∶K_2_NaScF_6_	430	1.4	630	0.7	760	-	1.3	285	[3]
Cr∶GSGG	488	5.1	647.1	3.3	777	-	0.75	114	[7]
Cr∶LiCaAlF_6_	425.5	-	625	-	780	2000 cm^−1^	1.23	175	[4]
Cr∶LaSc_3_(BO_3_)_4_								17.0	[8]
E//x	457	1.18	654	1.68	948	200 nm	6.13		
E//y	456	1.72	655	1.01	948	210 nm	5.83		
E//z	458	1.32	655	0.81	948	280 nm	4.33		
Cr∶KAl(MoO_4_)_2_								33.0	[10]
σ-Polarization	480	8.44	669	3.72	823	146 nm	2.74		
π-Polarization	481	5.03	668	2.25	823	135 nm	2.93		
Cr∶RbAl(MoO_4_)_2_	479	10.15	668	5.81	822.7	138 nm		25.7	[27]
Cr∶CsAl(MoO_4_)_2_	481	5.05	670	3.06	818	147	4.27	21.0	[28]
Cr∶ MgMoO_4_								1.0	This work
E//**N_p_**	489	10.27 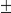 0.02	703	12.67 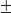 0.02	919	182 nm	107.56 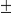 0.25		
E//**N_g_**	493	12.94 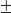 0.02	705	7.89 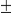 0.02	963	188 nm	115.69 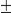 0.25		
E//**N_m_**	508	10.71 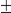 0.02	711	10.64 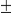 0.02	956	176 nm	124.63 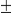 0.28		
